# FIT-Binding Proteins and Their Functions in the Regulation of Fe Homeostasis

**DOI:** 10.3389/fpls.2019.00844

**Published:** 2019-06-26

**Authors:** Huilan Wu, Hong-Qing Ling

**Affiliations:** ^1^The State Key Laboratory of Plant Cell and Chromosome Engineering, Institute of Genetics and Developmental Biology, Chinese Academy of Sciences, Beijing, China; ^2^College of Life Sciences, University of Chinese Academy of Sciences, Beijing, China

**Keywords:** Arabidopsis, plant nutrition, iron, FIT, FIT-binding protein, transcriptional regulation

## Abstract

Iron, as an essential micronutrient, is required by all living organisms. In plants, the deficiency and excess of iron will impair their growth and development. For maintaining a proper intracellular iron concentration, plants evolved different regulation mechanisms to tightly control iron uptake, translocation and storage. FIT, a bHLH transcription factor, is the master regulator of the iron deficiency responses and homeostasis in Arabidopsis. It interacts with different proteins, functioning in controlling the expression of various genes involved in iron uptake and homeostasis. In this review, we summarize the recent progress in the studies of FIT and FIT-binding proteins, and give an overview of FIT-regulated network in iron deficiency response and homeostasis.

## Introduction

Iron plays an essential role in a variety of biologic processes in plants, such as photosynthesis, respiration, nitrogen fixation and vitamin synthesis (Schmidt, 2003; Hansch and Mendel, 2009; Jeong and Guerinot, 2009; Zhao et al., 2011). Although iron is the fourth most abundant element in the earth’s crust, the deficiency of iron is one of the factors mostly limiting plant growth because it is mainly present as insoluble form Fe^3+^ and not available for plants in soil, especially in the neutral and basic soil. In order to overcome iron deficiency, plant developed the reduction-based (strategy I) and chelation-based (strategy II) mechanisms to mobilize and enhance iron uptake from soil (Marschner et al., 1986; Römheld and Marschner, 1986). All dicots and non-graminaceous plants use the strategy I mechanism whereas grasses apply the strategy II. Under iron limitation condition, the strategy I plants secrete proton and phenolic compounds from roots to rhizosphere for increasing the solubility of ferric iron (Santi and Schmidt, 2009; Fourcroy et al., 2014); the mobilized ferric iron will then be reduced to ferrous iron on root surface by the ferric chelate reductase, such as Ferric Reduction Oxidase2 (FRO2) (Robinson et al., 1999); finally, the reduced ferrous iron will be transported into root epidermal cells by the metal transporter Iron-Regulated Transporter1 (IRT1) (Vert et al., 2002). The strategy II plants (grasses) secrete phytosiderophores (PS) from roots into soil to chelate Fe(III), and the PS-Fe(III) complex will be transported into root cells by YS1 under the stress of iron deficiency (Takagi et al., 1984; Mori and Nishizawa, 1987; Curie et al., 2001). On the other hand, excessive iron can generate oxidative stress in cells and become toxic for plants. Therefore, the uptake, translocation and storage of iron in plants should be carefully regulated.

In strategy I plants, the expression of many genes is induced or upregulated at transcriptional level under iron limitation. In the processes, transcription factors are required and play important roles. *FER*, the first isolated regulatory gene of iron homeostasis from tomato, encodes a typical basic helix-loop-helix protein (Ling et al., 2002). The *FER*-null mutant *T3238fer*, which was caused by the insertion of a Ty1-copia-like element in the first exon of *FER* (Cheng et al., 2009), lost the whole iron deficiency responses. The expression of ferric-chelate reductase *LeFRO1* and ferrous transporter *LeIRT1* was not able to be upregulated in the mutant at the stress of iron deficiency (Ling et al., 2002; Li et al., 2004). The mutant showed severe chlorosis and died at young seedling stage under normal culture condition. FIT (AtbHLH29/FRU), a FER ortholog in Arabidopsis, was confirmed to enable complementation of the defective function in *T3238fer* (Yuan et al., 2005). The *FIT*-null mutant *fit-1* and *fit-3* generated by T-DNA insertion showed similar phenotypes as tomato *T3238fer*. The transcription of *FRO2* and *IRT1* in *fit-1* and *fit-3* was severely impaired (Colangelo and Guerinot, 2004; Jakoby et al., 2004). Microarray analysis showed that 72 genes upregulated under iron deficiency in wild type were completely or partially deregulated in the mutant *fit-1* (such as *AHA2*, *IRT1*, *IRT2, NRAMP1*, and *NAS1*), some of which were proposed to be its direct targets (Colangelo and Guerinot, 2004; Jakoby et al., 2004; Yuan et al., 2008; Ivanov et al., 2011; Sivitz et al., 2012; Wang et al., 2013). Recently, the list of FIT-regulated genes was expanded (Mai et al., 2016). As a key factor in the transcriptional regulation of Fe uptake and homeostasis, the expression of *FIT* is tightly regulated at transcriptional and posttranscriptional levels.

In this review, we summarize the recent progress in the studies of FIT and several of its interaction partners in *Arabidopsis thaliana* (a model plant of strategy I plants), and give an overview of FIT-related regulation pathway in iron deficiency responses and homeostasis of strategy I plants.

## Regulators Interacted With Fit in Initiating Iron Deficiency Responses

### Ib Subgroup of bHLH Transcription Factors

As shown in introduction, FIT, an ortholog of tomato FER, is a key transcription factor in the control of iron deficiency responses and homeostasis in *Arabidopsis thaliana* (Colangelo and Guerinot, 2004; Jakoby et al., 2004; Yuan et al., 2005). However, overexpression of *FIT* didn’t result in obvious phenotypical and physiological alterations, compared to wild type (Colangelo and Guerinot, 2004; Jakoby et al., 2004; Yuan et al., 2005; Meiser et al., 2011; Sivitz et al., 2011; Gratz et al., 2019). Based on these, it was suggested that FIT is essential, but it alone is not sufficient for the activation of iron deficiency responses and the effective iron uptake system in Arabidopsis. Indeed, Yuan et al. (2008) and Wang et al. (2013) revealed that the Ib subgroup of bHLH transcription factors bHLH038, bHLH039, bHLH100, and bHLH101 were able to interact with FIT to form the heterodimer of FIT/bHLH38, FIT/bHLH39, FIT/bHLH100, and FIT/bHLH101 in plant cells. Further, they did the transcriptional activation assay, and showed that the co-expression of *FIT* with one of the Ib subgroup of bHLH transcription factors enabled to activate *GUS* expression driven by either the *FRO2* or the *IRT1* promoter in yeast cells, suggesting that the formed heterodimer complex could bind to the promoter of iron uptake genes *FRO2* and *IRT1*, and directly activated their expression (Yuan et al., 2008; Wang et al., 2013). The expression of the four Ib bHLH genes was dependent on iron status, and strongly upregulated in root and leaf under iron starvation (Vorwieger et al., 2007; Wang et al., 2007). The plants double overexpressing *FIT* with *bHLH38*, *bHLH39*, *bHLH100*, or *bHLH101* changed the expression patterns of *FRO2* and *IRT1* from induced to a constitutive expression regardless iron status (Yuan et al., 2008; Wang et al., 2013). Phenotypically, the transgenic plants revealed a higher ferric-chelate reductase activity in root and accumulated more iron in shoot than wild type and the plants overexpressing of *bHLH38*, *bHLH39*, *bHLH100*, or *bHLH101* alone. The four Ib bHLH genes have a high sequence identity, and function redundantly in the regulation of iron uptake and homeostasis. Therefore, the single and double mutants of the four genes did not show obviously phenotypical and physiological alterations, whereas their triple mutant bHLH38/100/101 and bHLH39/100/101 exhibited sensitivity to iron limitation and chlorotic phenotype, compared to wild type (Wang et al., 2013). Additionally, they found that the four bHLH proteins possessed different significance in regulation of iron-deficiency responses and uptake (bHLH 39 > bHLH101 > bHLH38 > bHLH100). The similar results were also reported by Sivitz et al. (2012) and Maurer et al. (2014). They did not observed an obvious effect on the expression of iron acquisition genes in double knocking out-mutant *bhlh100bhlh101* and triple knocking-out mutant *bhlh39bhlh100bhlh101* (*3xbhlh*).

In addition to the activation function, Cui et al. (2018) recently reported that Ib bHLH proteins have a stabilization function for FIT in plant cells. They detected abundant FIT protein accumulation in roots of plants overexpressing *FIT* and *bHLH38*, but not in the roots of plants overexpressing *FIT* alone under iron-sufficient conditions. Further, they also observed that the roots of *bHLH38*-overexpressing plants accumulated much more FIT under iron sufficiency and protected JA-induced FIT degradation under JA treatment, compared to wild type. Based on the data, they speculated that the Ib subgroup of bHLH transcription factors might play a role in the protection of FIT from degradation. Alternatively, the phenomenon may be caused by higher expression of *FIT* in bHLH38-overexpressing plants and *FIT* and *bHLH38* double overexpressing plants. However, as reported by Yuan et al. (2008), over expression of *bHLH038* did not change the expressional level of *FIT*, compared with wild type. Anyway, more experiments are needed to confirm it.

The expression of the Ib bHLH genes was controlled genetically and epigenetically at transcription level. Shk1 binding protein 1 (SKB1/AtPRMT5), which catalyzes the symmetric dimethylation of histone H4R3 (H4R3sme2), was identified to be involved in iron homeostasis in Arabidopsis, while the SKB1 lesion mutant exhibited higher iron content in shoots and more tolerance to iron deficiency, compared to wild type (Fan et al., 2013). The expression of *SKB1* was not dependent on iron, but the level of H4R3sme2 mediated by SKB1 was clearly related to iron status in plants. Chromatin immunoprecipitation (ChIP) and genome-wide ChIP-seq results showed that SKB1 associated with the chromatin in the promoter region of *bHLH38*, *bHLH39*, *bHLH100* and *bHLH101*, and symmetrically dimethylated histone H4R3 (Fan et al., 2013). Iron deficiency may cause an increase in the disassociation of SKB1 from chromatin of the four Ib bHLH genes and resulted in a decrease of H4R3sme2 level, thereby elevating their transcription and enhancing iron uptake.

At genetic control, a recent report showed that the expression of *bHLH38*, *bHLH39*, *bHLH100*, and *bHLH101* was positively regulated by bHLH034, bHLH104, bHLH105, and bHLH115, which are classified as the IVc subgroup of bHLH transcription factors. The mutants of IVc bHLH transcription factors displayed sensitivity to iron deficiency, including reduced root length, chlorotic leaves, and decreased ferric chelate reductase activity (Zhang et al., 2015; Li et al., 2016; Liang et al., 2017). Under both Fe-sufficient and Fe-deficient conditions, the transcript levels of *bHLH38*, *bHLH39*, *bHLH100*, and *bHLH101* were always lower in the mutants of IVc bHLH transcription factors than in wild-type plants. Transient expression assays and ChIP experiments confirmed that IVc bHLH transcription factors were able to directly activate the promoters of *bHLH38*, *bHLH39*, *bHLH100*, and *bHLH101* (Zhang et al., 2015; Li et al., 2016; Liang et al., 2017).

### EIN3 and EIL1

As known for a long time, ethylene as a phytohormone plays important roles in iron deficiency responses. Its molecular mechanism has been elucidated recently. Lingam et al. (2011) reported that EIN3 (Ethylene Insensitive 3) and EIL1 (EIN3-Like 1), two transcription factors involved in the ethylene signaling pathway, can interact with FIT, and showed that the interaction of FIT with EIN3 and EIL1 was required for FIT accumulation, and contributes to the expression of FIT-downstream genes. Loss function of *EIN3* and *EIL1* led to the significant reduction of FIT accumulation even under iron deficiency condition.

### MED16 and MED25

During the transcription process, Mediator, a large protein complex comprising more than 20 subunits, is required and functions as a bridge connecting the RNA polymerase II (Pol II) complex and specific transcriptional activators (Asturias et al., 1999). Through interaction with specific transcription activators, Mediator coordinates and transfers the developmental and environmental signals to the transcriptional machinery to regulate the expression of corresponding genes (Bäckström et al., 2007; Mathur et al., 2011; Zhang et al., 2012). MED16 (Mediator subunit 16) and MED25 (Mediator subunit 25), organized in the tail module of the Mediator, have been recently identified to be involved in the regulation of Fe-dependent gene expression (Yang et al., 2014; Zhang et al., 2014). Loss of MED16 or MED25 led to a declined expression of some iron deficiency response genes, such as *FRO2*, *IRT1* and *AHA2*, under iron deficiency condition. The mutant *med16* and *med25* showed hypersensitivity to the stress of iron limitation. Zhang et al. (2014) demonstrated that MED16 functioned in the interaction with FIT to enhance the expression of FIT-dependent genes by recruiting FIT-bHLH complex to their promoters. Additionally, Yang et al. (2014) found that MED25 was able to interact with MED16, EIN3 and EIL1, which may play some roles in FIT stabilization.

### CIPK11

Cytosolic Ca^2+^ as the second messenger is an important signaling molecule to transduce developmental and environmental cues to activate physiological processes in plants. Recently, Tian et al. (2016) and Gratz et al. (2019) used the method of fluorescence resonance energy transfer (FRET), and observed an increased cytoplasm Ca^2+^ concentration in primary roots under iron deficiency. Further, they physiologically characterized the loss-of-function mutants of *CIPK11*, *CIPK23*, and *CBL1*/*CBL9* on iron deficiency medium, and demonstrated that calcium-dependent CBL-CIPK pathway was involved in response to iron deficiency in Arabidopsis. With yeast-two-hybrid and BiFC assay, Gratz et al. (2019) confirmed that CIPK11 was positively involved in iron deficiency response through directly interaction with FIT. *CIPK11* transcript abundance was significantly elevated under iron deficiency, and its expression pattern was similarly to the *FIT* in the early differentiation root zone, in which iron uptake occurs predominantly (Gratz et al., 2019). Loss of CIPK11 function resulted in the partial inactivation of the iron deficiency responses and reduced the iron content in seed. *In vitro* assay displayed that CIPK11 as a kinase enabled phosphorylation of FIT at Ser271 amino acid (Gratz et al., 2019). Further, using a series of molecular methods including *fit-3* mutant complementation assay, protein homo- and hetero-dimerization assay in yeast and planta, transcription self-activation capacity assay and fluorescence recovery after photobleaching analysis (Gratz et al., 2019), they revealed that phosphorylation of Ser272 amino acid modulated FIT nuclear accumulation, homo-dimerization, interaction with bHLH039 and transcriptional activity, and proposed that FIT phosphorylation status determines its activity.

## Regulators Interacted With Fit in Repressing Iron Deficiency Responses

### ZAT12

As known, H_2_O_2_ as a signal is involved in many abiotic stresses (Mittler and Blumwald, 2015). Increase in H_2_O_2_ accumulation was observed in plant roots under the deficiency of potassium, nitrogen, phosphorus and iron (Ranieri et al., 2001; Shin et al., 2005; Sun et al., 2007). Recently, Le et al. (2016) reported that zinc finger of *Arabidopsis thaliana* 12 (ZAT12) functioned in the negative regulation of plant responses to prolonged iron deficiency stress. ZAT12 is a marker of abiotic stress and its expression is induced by oxidative stress. Thus, a molecular connection between Fe deficiency and the oxidative stress response was illustrated. *ZAT12* was expressed mainly in the root early differentiation and elongation zone, and responded to iron deficiency in the late stage, compared with Fe sufficiency. Using yeast-two-hybrid approach and BiFC assay in plant, they demonstrated that the EAR motif of ZAT12 interacted with FIT-C terminus. Based on the result that *FIT* expression was down-regulated and *ZAT12* expression was up-regulated under iron deficiency with H_2_O_2_ stress, ZAT12 should be a repressor of FIT. The formation of FIT-ZAT12 complexes in root cells would inactivate the FIT under prolonged Fe deficiency condition.

### DELLA

Iron deficiency will trigger obvious changes in root architecture, such as reduced primary root length and increased frequency of root hairs, as well as high affinity iron uptake mechanism. Exogenous gibberellin (GA) treatment has been shown to promote the expression of *IRT1*, *FRO2*, *bHLH38*, and *bHLH39* in the GA-deficient mutant *ga3ox1ga3ox2* (the double-knockout mutant of GA biosynthetic gene *GA3OX1* and *GA3OX2* which are involved in later steps of the gibberellic acid biosynthesis), although the expression of *FIT* was not induced by the application of GA (Matsuoka et al., 2014). Recently, Wild et al. (2016) demonstrated that GA signaling was involved in the iron deficiency response by interaction of DELLA with protein FIT, bHLH38, and bHLH39. DELLA-FIT interaction did not interfere with the formation of FIT-Ib bHLH heterodimer, but impaired the binding capacity of the FIT-Ib bHLH complex to corresponding promoters. DELLA protein exists in all tissues of root under normal condition. Under iron deficiency, DELLA protein will accumulate in the root meristem to repress the root growth, and degrade in the epidermis cells of the differentiation zone, relieving FIT from the DELLA-FIT complex for activating the expression of iron uptake genes (Wild et al., 2016).

### FBP

Under iron deficiency, plants will upregulate the expression of Fe transporter *IRT1* in strategy I plants. Due to the low substrate specificity of IRT1 protein, other divalent transition metals, such as Zn, will be transported into root cells. Thus, the excessive Zn will be acquired into root cells from the environment, and result in toxicity to the plant under the condition of iron limitation. To keep the Zn balance and detoxify in cells, plant will up-regulate the expression of some genes related to sequestration and chelation of Zn. Chen et al. (2018) used yeast-two-hybrid approach and identified a novel FIT-binding protein (FBP), which is involved in the distribution and balance of Fe and Zn homeostasis. They confirmed that FBP sequestered FIT to obstruct the heterodimer formation of FIT with Ib bHLH protein in the root stele and negatively regulated the expression of *NAS1, NAS2*, and *NAS4* genes to balance the Fe and Zn homeostasis in Arabidopsis. FBP transcript abundance significantly reduced along with increased Zn concentration. The plants with loss function of *FBP* displayed the elevated expression of *NAS* genes, and tolerance to excessive Zn stress (Chen et al., 2018). Further, they did DNA-protein binding and transcriptional activation analysis, and confirmed that FBP inhibited the binding of FIT-Ib bHLH heterodimer to the promoters of *NAS* genes. The negative regulation caused by sequestration of FIT is restricted in the root stele, but not in epidemic cells, because *FBP* only expressed in the root stele. Considering that FIT protein accumulation was comparable in roots of *FBP*-null mutant *fbp* with wild type under high Zn condition (Chen et al., 2018), the main function of FBP looks like to inactivate FIT by blocking heterodimer formation with Ib bHLH proteins, but not for its degradation.

### IVa bHLH Transcription Factors

Jasmonic acid (JA) as a negative factor represses the upregulated expression of *FIT*, *IRT1* and *FRO2* under iron deficiency (Maurer et al., 2011). The depressed regulation mechanism has been elucidated recently. Cui et al. (2018) identified the IVa subgroup of bHLH transcription factors (bHLH18, bHLH19, bHLH20, and bHLH25), which are involved in the repressed regulation of iron deficiency responses under JA present. The four IVa bHLH genes expressed mainly in roots and responded to JA. Characterization of the single, double, triple and quadruple mutants of the four IVa bHLH genes revealed that the four bHLH transcription factors functioned redundantly in the JA-mediated inhibition of iron acquisition in Arabidopsis. Immunoblot analysis showed that bHLH18, bHLH19, bHLH20, and bHLH25 interacted with FIT and promoted its degradation mediated by JA under iron deficiency. Based on that the plants overexpressing Ib bHLH genes can alleviate JA-mediated FIT protein degradation, and exhibit more tolerance to iron deficiency under JA present, Cui et al. (2018) speculated that the main function of the four IVa bHLH proteins might be to compete with Ib bHLH proteins to bind FIT and stimulates its degradation. Further, they demonstrated that JA-promoted degradation of the FIT protein is through the 26S proteasome pathway. Additionally, they showed that MYC2 and JAR1, critical components of the JA signaling pathway, differentially regulated the expression of *bHLH18*, *bHLH19*, *bHLH20*, and bHLH25 to modulate FIT protein accumulation, and MYC2 depressed the expression of *FIT* and the four Ib bHLH genes at transcription level. Taken together, upregulated expression of IVa bHLH genes and downregulated expression of *FIT* and Ib bHLH genes under JA present result in lower accumulation of activators FIT and Ib bHLH proteins, consequently downregulating the expression of the iron-uptake genes *IRT1* and *FRO2*. Considering that JA is an important integrator that balances plant growth and defense responses, the JA-mediated suppression of iron acquisition in plants should play some important roles in defense responses against biotic and abiotic stresses to promote plant survival.

## Conclusion

**FIGURE 1:**
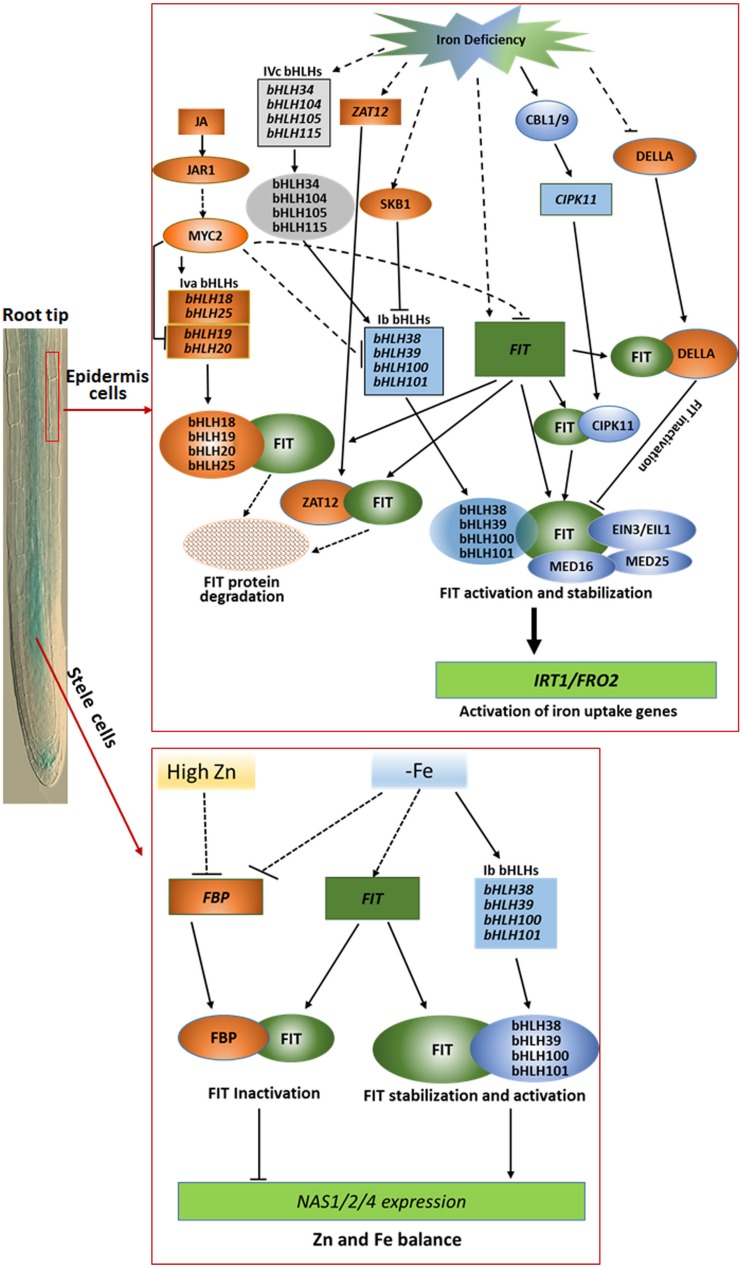
Outline of the regulation networks and functions of FIT-binding proteins in iron deficiency responses and homeostasis in root epidermis cells (upper part) and stele cells (lower part). In root epidermal cells, Ib bHLHs, MED16, MED25, EIN3/EIL1, and CIPK11 as positive modulators interacts with the key regulator FIT, functioning in activation of the expression of iron acquisition genes such as *FRO2* and *IRT1* under iron limitation condition, whereas DELLA, ZAT12, and IVa bHLHs as negative modulators compete with Ib bHLHs to bind FIT for negative regulation of FIT activity for avoidance of excessive iron uptake. In stele cells, FBP as a negative regulator sequestered FIT to obstruct the heterodimer formation of FIT with Ib bHLH protein to downregulate the expression of *NAS1*, *NAS2*, and *NAS4* for balancing Fe and Zn homeostasis in Arabidopsis under low iron or high Zn stress condition. The rectangles represent transcripts of the corresponding genes, and the ovals mean proteins indicated. The blue and orange color depicted positive and negative regulators, respectively. The solid and dotted lines separately indicate the known and unknown regulation processes.

As described above, the proteins encoded by 15 different genes have been confirmed to bind FIT during last several years in Arabidopsis. Their functions can be generally summarized into three categories, and outlined in Figure 1. The first group contains the four Ib bHLH proteins, CIPK11, MED16, EIN3, and EIL1. The eight proteins as positive regulators bind to FIT, and function as stabilizer and activator of FIT in iron deficiency responses and homeostasis. The second one is the four IVa bHLH proteins mediated by JA signal pathway and ZAT12. They play as negative regulators to enhance the degradation of FIT protein via interaction with FIT. The last group includes DELLA and FBP, which compete with Ib bHLH proteins to bind FIT, and inactivate its functions. These findings obviously reveal a new insight for understanding the regulation mechanisms of iron uptake and homeostasis in strategy I plants. Although a great progress has been made in studying the molecular regulation mechanisms of iron deficiency responses and homeostasis in strategy I plants in the past two decades, there are still many questions that are not answered. For example, iron uptake and homeostasis are tightly regulated dependent on iron status in plants, how does plant sense the iron status in cells and in environment, and how is the iron signal transduced in plants? As shown in Figure 1, some proteins, such as DELLA and FBP, involved in iron deficiency responses and homeostasis function only in a specific region or cell type of root, we still do not know what is the molecular mechanism(s) accurately controlling their spatial expression and functional patterns. As a key regulator, expression of *FIT* is controlled at transcriptional and posttranscriptional level. However, we do still not well know the regulation mechanisms.

## Author Contributions

Both authors listed have made a substantial, direct and intellectual contribution to the work, and approved it for publication.

## Conflict of Interest Statement

The authors declare that the research was conducted in the absence of any commercial or financial relationships that could be construed as a potential conflict of interest.
